# Relationship Between Sonographic Findings, Fine Needle Aspiration, and Histopathological Findings of Post-thyroid Surgery

**DOI:** 10.7759/cureus.46133

**Published:** 2023-09-28

**Authors:** Marcos Calzado Capobianco, Omar W Ebrahim Ibrahim, Alberto J Bonnet Ortiz, Yousef S Ebrahim Ibrahim, Angel Campusano, Hamid Feiz, Ammar Ibrahim

**Affiliations:** 1 Research, National Institute of Diabetes, Nutrition, and Endocrinology (INDEN), Santo Domingo, DOM; 2 Medicine, Universidad Iberoamericana (UNIBE), Santo Domingo, DOM; 3 Research, Universidad Iberoamericana (UNIBE), Santo Domingo, DOM; 4 Internal Medicine, Wellington Regional Medical Center, Wellington, USA; 5 Surgery, National Institute of Diabetes, Nutrition, and Endocrinology (INDEN), Santo Domingo, DOM

**Keywords:** fine needle aspiration, malignant tumors, benign tumors, final biopsy, ultrasound, thyroid surgery

## Abstract

Background: Thyroid diseases can affect various bodily functions and often go unnoticed. Tools such as sonography and fine needle aspiration (FNA) puncture are necessary to diagnose diseases that require surgical intervention. These tools help identify signs of malignancy or benignity and obtain further data to guide therapeutic decisions. This study aims to validate the relationship between sonographic results, FNA, and final thyroid pathology. This research describes the level of correlation between sonographic findings and FNA, the sonographic and final pathology reports, and the FNA and final pathology reports. Additionally, this research aims to identify the most common diagnoses in the final pathology.

Methods: A retrospective descriptive observational study was carried out with a sample of 95 patients who underwent thyroid surgery at the National Institute of Diabetes, Endocrinology, and Nutrition (INDEN), Dominican Republic, in 2019 to determine whether a relationship exists between the sonography findings, FNA, and the final pathology in surgical thyroid pathologies.

Results: A total of 95 patients were studied. The success rate of the sonography results compared with the benign final biopsy result was 100% and 45.9% with the malignant final biopsy result. The success rate of the fine needle biopsy results was 95.9% for the benign final biopsy and 28% for the malignant final biopsy. Of the malignant final biopsy reports, 84.6% were papillary carcinomas, 7.7% were follicular carcinomas, and 7.7% were medullary carcinomas.

Conclusions: The relationship between the sonographic results, FNA, and histopathological findings of surgical thyroid diseases is validated. The sonographic findings are specific for diagnosing benignity and malignancy. A fine needle biopsy is useful for diagnosing benignity, and the final biopsy is the standard for confirming both benign and malignant pathologies.

## Introduction

Around 300 million people worldwide suffer from thyroid disease. Surgical pathologies of the thyroid form a significant percentage of all thyroid diseases, including benign and malignant tumors. According to the American Thyroid Association, 60% of people with thyroid disease are unaware of their condition [1]. The Dominican Republic is vulnerable to these diseases due to notable risk factors, such as obesity and iodine deficiency, and a generally low level of health education, one of the lowest in Latin America. These issues highlight the need to conduct research to improve the detection and diagnosis of surgical thyroid diseases in this country.

Since several thyroid pathologies are similar, it is important to distinguish between them. Thyroid sonography and fine needle aspiration (FNA) puncture are tools used for detection and distinction and play a vital role in managing the disease and determining whether surgical intervention is necessary. Evaluating thyroid pathologies can be tedious if existing tools are not utilized fully. The data provided by each tool can be overlooked and deemed unimportant. However, the data obtained using a labeled tool can be sufficient to rule out or confirm malignancy [2], aiding the decision of whether to approach the disease surgically or clinically and avoiding unnecessary tests and procedures. This study addresses this problem by investigating the relationships between sonographic findings, FNA, and final pathology following thyroid surgery undertaken at the National Institute of Diabetes, Nutrition, and Endocrinology (INDEN) in 2019.

This research was conducted at INDEN due to its high flow of patients with endocrinological problems, with thyroid pathology responsible for a considerable percentage. The center performed approximately two to three thyroid operations per week. Samples were taken from patients who underwent the following investigated procedures in 2019: sonography, FNA puncture or biopsy, surgical resection of the gland (partial or total), and final histopathology.

This work's principal aim is to validate the relationship between sonographic results, FNA, and final thyroid pathology. Hence, this study investigates the level of correlation between sonographic findings and FNA, the sonographic and final pathology reports, and the FNA and final pathology reports. Additionally, this research identifies the most common diagnoses in the 2019 final pathology results and the sociodemographic variables associated with the most adverse types of neoplasms.

## Materials and methods

Ethics approval and consent to participant

The study data were obtained from the registered records of all patients who underwent thyroid surgery in 2019. The ethical principles and criteria were prioritized to ensure no individuals providing information were offended or harmed. This research was subject to approval by the ethics committees of the Universidad Iberoamericana (UNIBE) and INDEN.

Study population

The study population included all patients who underwent thyroid surgery at INDEN between January 1 and December 31, 2019, in Santo Domingo, Dominican Republic. A non-probabilistic convenience sample, comprising the total number of patients who underwent the procedure, was used. Observations and analysis of the clinical records of patients who attended an endocrinology and surgery consultation and subsequently underwent thyroid surgery were performed. Data were collected using indirect observation. A total of 95 patients (86 females and nine males) who underwent sonography, thyroid surgery, and a final biopsy participated in the study. Ninety-one patients underwent FNA. Fifty-two patients were from the urban region, while 43 were from rural areas.

Ultrasound evaluation

High-resolution ultrasound data were analyzed using the Thyroid Imaging Reporting and Data Systems (TI-RADS) classification and scored, as demonstrated in Table 1 [3,4]. Since not all sonography reports give the TI-RADS, sonographic descriptions were used to extrapolate this classification.

**Table 1:** TI-RADS classification Ultrasound data on suspicious nodule features: solid, hypoechoic, microcalcifications, irregular shape and margins (infringement, lobulation), taller-than-wide shape, uneven acoustic halo with rear attenuation, infringement of the thyroid envelope, rich blood flow within the nodule, abnormally shaped lymph nodes. TI-RADS = Thyroid Imaging Reporting and Data Systems

TI-RADS Grade	Evaluation	Description of ultrasound pattern	Score
1	Negative	Normal thyroid	
2	Benign	Liquid, mixed, regular shape, well-defined margin	1
3	Probably benign	No suspicious ultrasound features	2
4	Probably malignant	Solid, hypoechoic, microcalcifications, poor margins, defined/lobulated, aspect ratio >1 (taller-than-wide shape)	
4A	Low suspicion of malignancy	One suspicious ultrasound feature	3
4B	Intermediate suspicion of malignancy	Two suspicious ultrasound features	4
4C	Moderate concern but not classic for malignancy	Three or four suspicious ultrasound features	5
5	Highly suggestive of malignancy	More than 4 suspicious ultrasound features, including microcalcifications and lobulated, particularly	6
6	Malignant	Biopsy-proven malignancy	

FNA biopsy

The Bethesda system for reporting thyroid cytopathology and scoring (Table 2) was used to report pathological findings in thyroid nodules after FNA [3,5].

**Table 2:** Bethesda system

Diagnostic Category	Score
Category 1: Nondiagnostic or unsatisfactory	1
Category 2: Benign	0
Category 3: Atypia of undetermined significance of follicular lesion of undetermined significance	2
Category 4: Follicular neoplasm or suspicious for a follicular neoplasm	3
Category 5: Suspicious for malignancy	4
Category 6: Malignant	6

Statistical analysis

The collected information was organized using tables and graphs corresponding to the results obtained concerning the study variables. Electronic data processing was performed using Microsoft Word and Excel.

## Results

**Figure 1:**
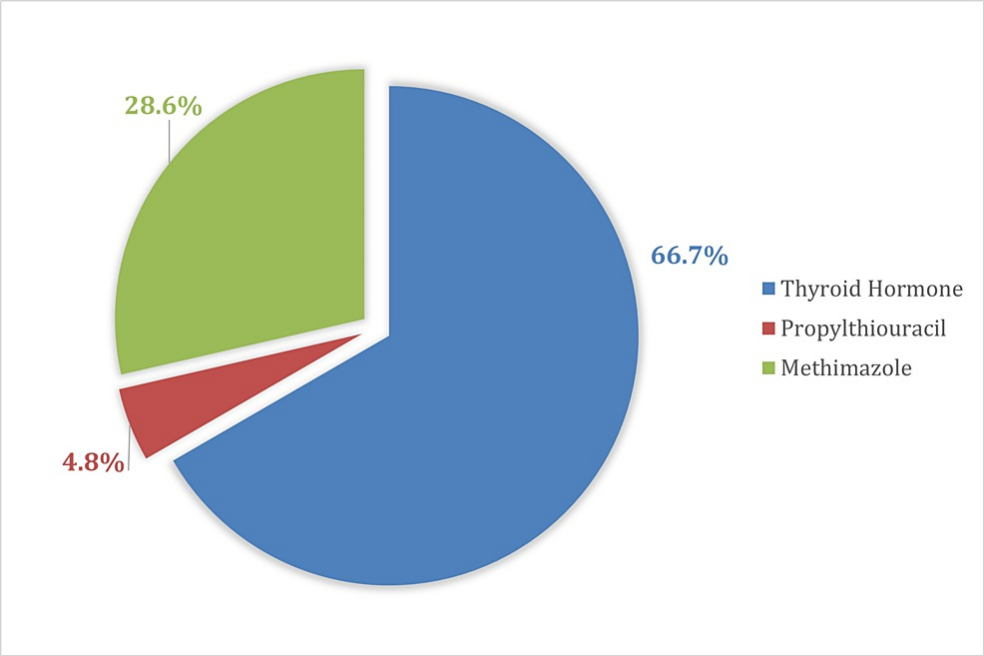
Distribution of the sample according to clinical characteristics

In total, 95 patients (86 females and nine males) underwent thyroid surgery. No patients were under 18 years old; 89 patients were between 18 and 71 years old, and six patients were older than 71 years. Twenty-one of the patients studied had prior drug treatment, of which 14 (66.7%) used a thyroid hormone, six (28.6%) used methimazole, and one (4.8%) used propylthiouracil (Figure 1).

Regarding previous surgical history, the sample’s distribution reveals that only five of the 95 patients had previously had thyroid surgery, as indicated in Table 3.

**Table 3:** Distribution of the sample according to previous surgical history

Previous thyroid surgery	Values	Percentage
Yes	5	5.3%
No	90	94.7%

**Figure 2:**
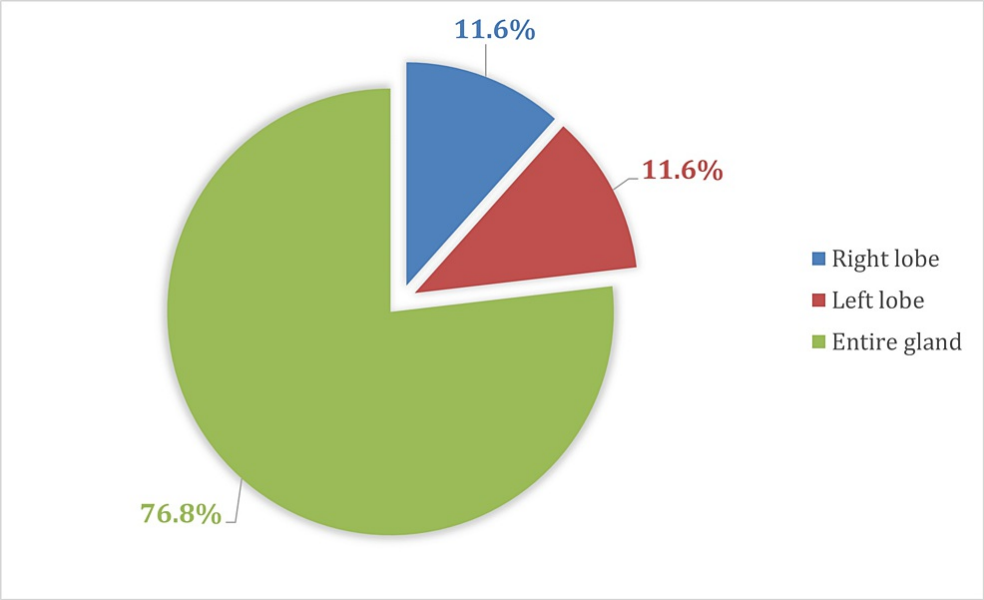
Distribution of the sample according to the affected thyroid area

Figure 2 reveals that 73 patients (76.8%) had entire thyroid involvement, 11 patients (11.6%) had right lobe involvement, and the remaining 11 patients (11.3%) had left lobe involvement.

According to the TI-RADS sonography classification, 6.3% were classed as TI-RADS I, 13.7% were classed as TI-RADS II, 35.8% were classed as TI-RADS III, 27.4% were classed as TI-RADS IV, and 16.8% were classed as TI-RADS V (Table 4).

**Table 4:** Distribution of the sample according to the TI-RADS sonography classification TI-RADS I: negative; TI-RADS II: benign; TI-RADS III: probably benign; TI-RADS IV: probably malignant; TI-RADS V: highly suggestive of malignancy [3].

Classification (TI-RADS)	Values	Percentage
TI-RADS I	6	6.3%
TI-RADS II	13	13.7%
TI-RADS III	34	35.8%
TI-RADS IV	26	27.4%
TI-RADS V	16	16.8%

Four of the 95 patients did not undergo a FNA biopsy. Of the remaining 91 patients, 49 had a benign result, 25 had a malignant result, and 17 had a result of cellular atypia (see Table 5).

**Table 5:** Distribution of the sample according to the fine needle biopsy

Fine needle biopsy	Values	Percentage
Benign	49	53.8%
Malignant	25	27.5%
Cellular atypia	17	18.7%

Since four of the 95 patients did not undergo a fine needle biopsy (FNB), the sample size for the Bethesda classification was 91 patients. According to the Bethesda classification, none of the patients who underwent an FNB belong to class I, 49 belong to class II, 12 belong to class IIIa, two belong to class IIIb, two belong to class IV, 22 belong to class V, and four belong to class VI (Table 6).

**Table 6:** Distribution of the fine needle biopsy sample according to the Bethesda classification Bethesda I: non-diagnostic or unsatisfactory; Bethesda II: benign; Bethesda IIIa: atypia of undetermined significance; Bethesda IIIb: follicular lesion of undetermined significance; Bethesda IV: follicular neoplasm or suspicious for a follicular neoplasm; Bethesda V: suspicious for malignancy; Bethesda VI: malignant [3].

Biopsy classification (Bethesda)	Values	Percentage
Bethesda I	0	0%
Bethesda II	49	53.8%
Bethesda IIIa	12	13.2%
Bethesda IIIb	2	2.2%
Bethesda IV	2	2.2%
Bethesda V	22	24.2%
Bethesda VI	4	4.4%

According to the final biopsy, 80 patients had a benign result, while 13 had a malignant result. Two patients’ final biopsies required additional immunohistochemical studies to confirm whether the pathology was benign or malignant.

**Figure 3:**
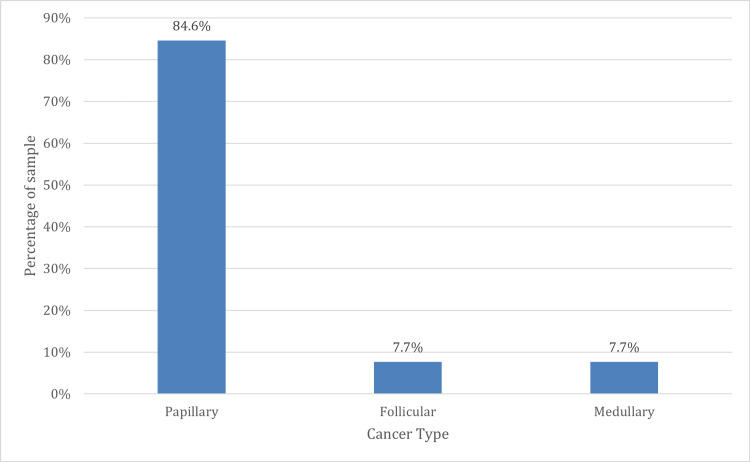
Distribution of the sample according to the histopathological type of the malignant tumor

According to the histopathological type of the malignant tumor, out of 13 patients, 11 cases (84.6%) were papillary adenocarcinoma, one (7.7%) was follicular adenocarcinoma, and one (7.7%) was medullary adenocarcinoma, as illustrated in Figure 3.

**Figure 4:**
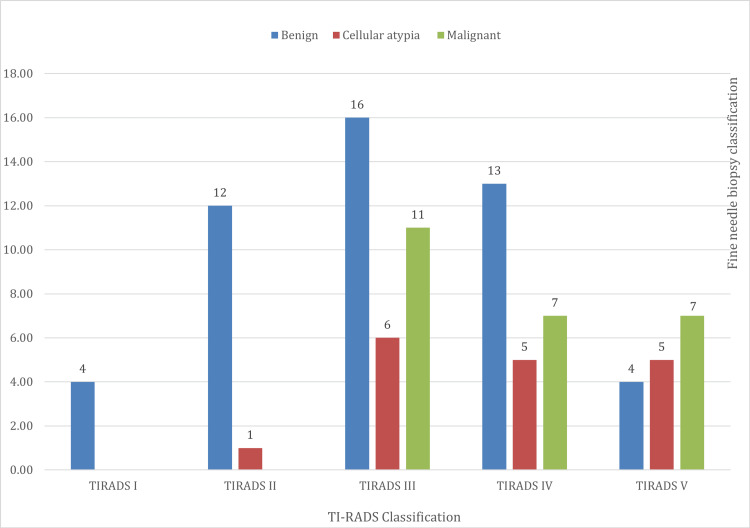
Relationship between the TI-RADS sonographic classification results and the fine needle aspiration (FNA) biopsy TI-RADS I: negative; TI-RADS II: benign; TI-RADS III: probably benign; TI-RADS IV: probably malignant; TI-RADS V: highly suggestive of malignancy [3].

Out of 91 patients, two of the six patients in TI-RADS I did not have an FNB; the remaining four patients all had benign FNB results. Of the 16 patients in TI-RADS V, the minority (just four patients) had a benign FNB result, indicating an inversely proportional relationship between the TI-RADS classification and the probability of having a benign FNB result (Figure 4).

The relationship between the sonography (TI-RADS) and Bethesda classifications reveals that no patients belong to B-I. The four patients classed as TI-RADS I belong to B-II. Of the 13 patients classed as TI-RADS II, 12 belong to B-II. Of the patients classed as TI-RADS III, 11 belong to B-V. Of the 25 patients classed as TI-RADS IV, 13 belong to B-II, three belong to B-IIIa, one belongs to B-IV, five belong to B-V, and three belong to B-VI. Of the 16 patients classed as TI-RADS V, four belong to B-II, five belong to B-IIIa, and six belong to B-V (Table 7).

**Table 7:** Relationship between the TI-RADS sonographic classification results and the Bethesda biopsy classification TI-RADS I: negative; TI-RADS II: benign; TI-RADS III: probably benign; TI-RADS IV: probably malignant; TI-RADS V: highly suggestive of malignancy [3]. B-I= Bethesda I: non-diagnostic or unsatisfactory; B-II= Bethesda II: benign; B-III= Bethesda IIIa: atypia of undetermined significance; B-IIIb= Bethesda IIIb: follicular lesion of undetermined significance; B-IV= Bethesda IV: follicular neoplasm or suspicious for a follicular neoplasm; B-V= Bethesda V: suspicious for malignancy; B-VI= Bethesda VI: malignant [3].

	B-I	B-II	B-IIIa	B-IIIb	B-IV	B-V	B-VI
TI-RADS I (4 patients)	0	4	0	0	0	0	0
TI-RADS II (13 patients)	0	12	1	0	0	0	0
TI-RADS III (33 patients)	0	16	3	2	1	11	0
TI-RADS IV (25 patients)	0	13	3	0	1	5	3
TI-RADS V (16 patients)	0	4	5	0	0	6	1

**Figure 5:**
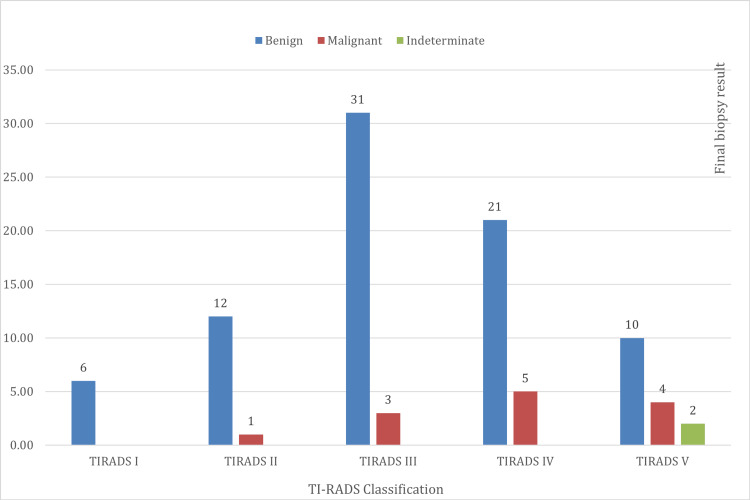
Relationship between the TI-RADS sonographic classification results and the final biopsy out of 95 patients TI-RADS I: negative; TI-RADS II: benign; TI-RADS III: probably benign; TI-RADS IV: probably malignant; TI-RADS V: highly suggestive of malignancy [3].

The relationship between the sonography and final biopsy results reveals that all six patients classed as TI-RADS I had a benign result. A proportional increase in the probability of a malignant result was evident between TI-RADS II and TI-RADS IV. Of the 16 patients in TI-RADS V, 10 had a benign result, four had a malignant result, and two had an indeterminate result (Figure 5).

**Figure 6:**
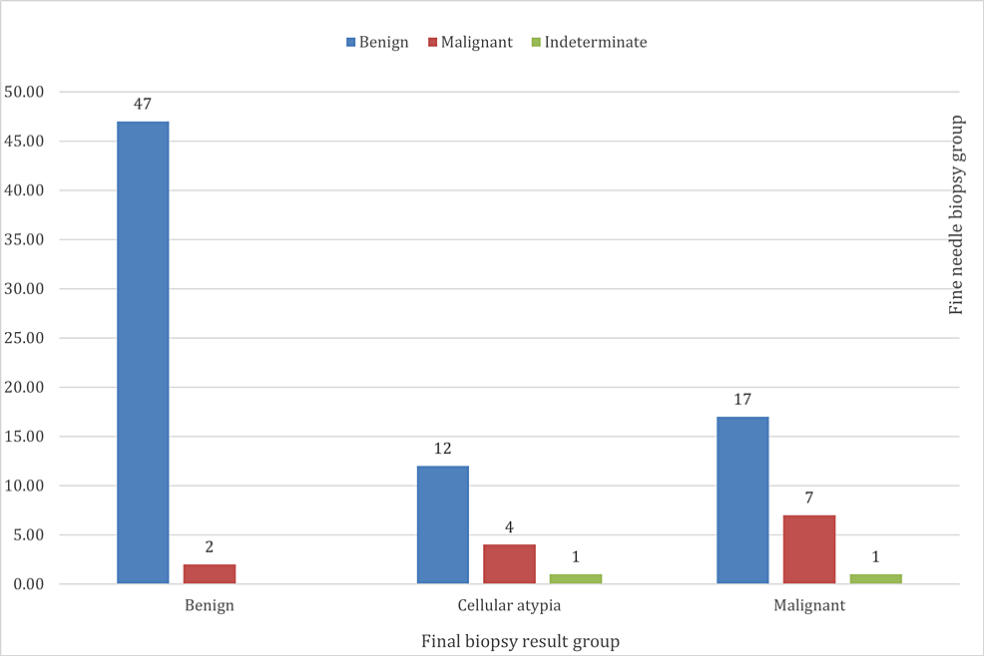
Relationship between FNA biopsy and final biopsy results

A disproportional relationship was observed between the FNB and 91 patients who received final biopsy results. This relationship indicates that most of the suspicious malignant cases reported by the FNB were identified as benign in the final biopsy result (Figure 6).

The relationship between the Bethesda biopsy classification and the final pathology biopsy results indicates that nine of the 12 patients with an IIIa classification had a benign final biopsy result. Of the 22 patients with a V classification, 16 had a benign final biopsy result, and five had a malignant final biopsy result. All four patients with a VI classification had a malignant final biopsy result (Table 8).

**Table 8:** Relationship between the Bethesda biopsy classification results and the final pathological biopsy B-I= Bethesda I: non-diagnostic or unsatisfactory; B-II= Bethesda II: benign; B-III= Bethesda IIIa: atypia of undetermined significance; B-IIIb= Bethesda IIIb: follicular lesion of undetermined significance; B-IV= Bethesda IV: follicular neoplasm or suspicious for a follicular neoplasm; B-V= Bethesda V: suspicious for malignancy; B-VI= Bethesda VI: malignant [3].

	Benign	Malignant	Indeterminate
B-I (0 patients)	0	0	0
B-II (49 patients)	47	2	0
B-IIIa (12 patients)	9	2	1
B-IIIb (2 patients)	1	1	0
B-IV (2 patients)	2	0	0
B-V (22 patients)	16	5	1
B-VI (4 patients)	4	0	0

## Discussion

In this study, 90.5% (n = 86) of the 95 patients were female, and 9.5% (n = 9) were male. In a study by Triantafillou et al. in Greece, of 1,113 patients who underwent FNA, females predominated at 81.1% with males at 18.9% [6], indicating similar demographic data suggesting that thyroid disease is predominant in females.

Sonographic findings using the TI-RADS criteria revealed that, in the study population (N = 95), 6.3% (n = 6) belonged to TI-RADS I (benign); 13.7% (n = 13) belonged to TI-RADS II (not suspicious for malignancy); 35.8% (n = 34) belonged to TI-RADS III (mild suspicion for malignancy); 27.4% (n = 26) belonged to TI-RADS IV (moderate suspicion for malignancy); and 16.8% (n = 16) belonged to TI-RADS V (highly suggestive of malignancy). A similar distribution, with most of the results classed as TI-RADS II and III, was reported in a study by Zhang et al.: in a sample of 3,980 thyroid nodules studied, 74.2% were classed as TI-RADS II, 11.7% as TI-RADS III, 13.5% as TI-RADS IV, and 0.6% as TI-RADS V [7].

The relationship between the sonography and final biopsy results indicates that all six patients classed as TI-RADS I had a benign result. Of the 13 patients classed as TI-RADS II, 92.3% (n = 12) had a benign result, while 7.7% (n = 1) had a malignant result. Of the 34 patients classed as TI-RADS III, 91.2% (n = 31) had a benign result, while 8.8% (n = 3) had a malignant result. Of the 26 patients classed as TI-RADS IV, 80.8% (n = 21) had a benign result, while 19.2% (n = 5) had a malignant result. One of the 16 patients classed as TI-RADS V did not have a final biopsy. Therefore, of the remaining 15 patients, 60% (n = 9) had a benign result, 26.7% (n = 4) had a malignant result, and 13.3% (n = 2) had an indeterminate result. These results are similar to the previously discussed study by Zhang et al., where the majority of TI-RADS II, TI-RADS III, and TI-RADS IV had benign results [7].

The Bethesda classification was determined through FNA or FNB in 91 patients. No patients were classed as B-I (Bethesda I), suggesting a non-diagnostic biopsy or insufficient material; 53.8% (n = 49) were classed as B-II (benign); 13.2% (n = 12) were classed as B-IIIa (atypia of undetermined significance); 2.2% (n = 2) were classed as B-IIIb (follicular lesion of undetermined significance); 2.2% (n = 2) were classed as B-IV (follicular neoplasm); 24.2% (n = 22) were classed as B-V (suspected malignancy); and 4.4% (n = 4) were classed as B-VI (malignant). In the study by Triantafillou et al. in Greece, "22.9% were characterized as non-diagnostic (B-I), 70.1% were diagnosed as benign (B-II), 3.1% were diagnosed as atypical/follicular lesion of undetermined significance (B-IIIa and B-IIIb), 0.9% were diagnosed as follicular neoplasm or suspicious for follicular neoplasm (B-IV), while 1.2% of cases were categorized as suspicious for malignancy (B-V) and 1.8% as malignant (B-VI)", demonstrating an increased rate of malignancy in the Dominican population in comparison with the Greek population [6].

The relationship between the FNB classification and final biopsy results reveals that of the 49 patients with a benign classification, 95.9% had a final biopsy result that was benign, and 4.1% had a final biopsy result that was malignant. The 17 patients with cellular atypia had the following final biopsy results: 70.6% were benign, 23.5% were malignant, and 5.9% were indeterminate. The 25 patients with malignant FNB had the following final biopsy results: 68% were benign, 28% were malignant, and 4% were indeterminate. In contrast, in a study by Lew et al., 46% of the patients undergoing a thyroidectomy had thyroid cancer in the final histopathology. Of the FNB results that were non-diagnostic due to an inconclusive result, 76% had benign histopathology, and 24% had malignant histopathology. Of the FNB results that were benign, 85% agreed with the final histopathology, while 8.6% were malignant. Of the FNA results that were malignant, 98% agreed with the final histopathology, while 2% were benign [8].

The final biopsy results revealed that 84.2% (n = 80) of the study population had a benign result, 13.7% (n = 13) had a malignant result, and 2.1% (n = 2) had an indeterminate result, indicating that this portion of the population required immunohistochemical studies to obtain a more accurate and definitive diagnosis. Of the 13.7% with a malignant result, 84.6% (n = 11) were diagnosed with papillary carcinoma, 7.7% (n = 1) with follicular carcinoma, and 7.7% (n = 1) with medullary carcinoma. In contrast, in a study by Anand et al., 71% of the patients who underwent surgery had a benign final biopsy result, while 29% had a malignant final biopsy result. Of the malignant cases, 17% were diagnosed with papillary carcinoma, the most common neoplasm in the study, while 6% were diagnosed with follicular carcinoma [9,10].

Among the main limitations encountered in this research work was COVID-19, as restrictions on hospital access due to high infection rates extended and complicated the data collection process. Additionally, not all ultrasound reports included the TI-RADS classification, so sonographic descriptions were used to extrapolate this classification. Moreover, not all patients had a final biopsy report, as pathologists sometimes deemed it necessary to conduct immunohistochemical studies to obtain the ultimate result.

## Conclusions

Comparing sonographic findings with FNB and the final biopsy demonstrated that this method is specific for diagnosing and differentiating benign and malignant thyroid neoplasms. The sonographic classification confirmed that the higher the TI-RADS classification, the higher the probability of malignancy in the FNB. The directly proportional relationship between the FNB and final biopsy was validated. Finally, this study demonstrated that the FNB is a reliable method of diagnosing benign thyroid pathologies but imprecise when diagnosing malignant pathologies.

## Figures and Tables

**Figure 7:**
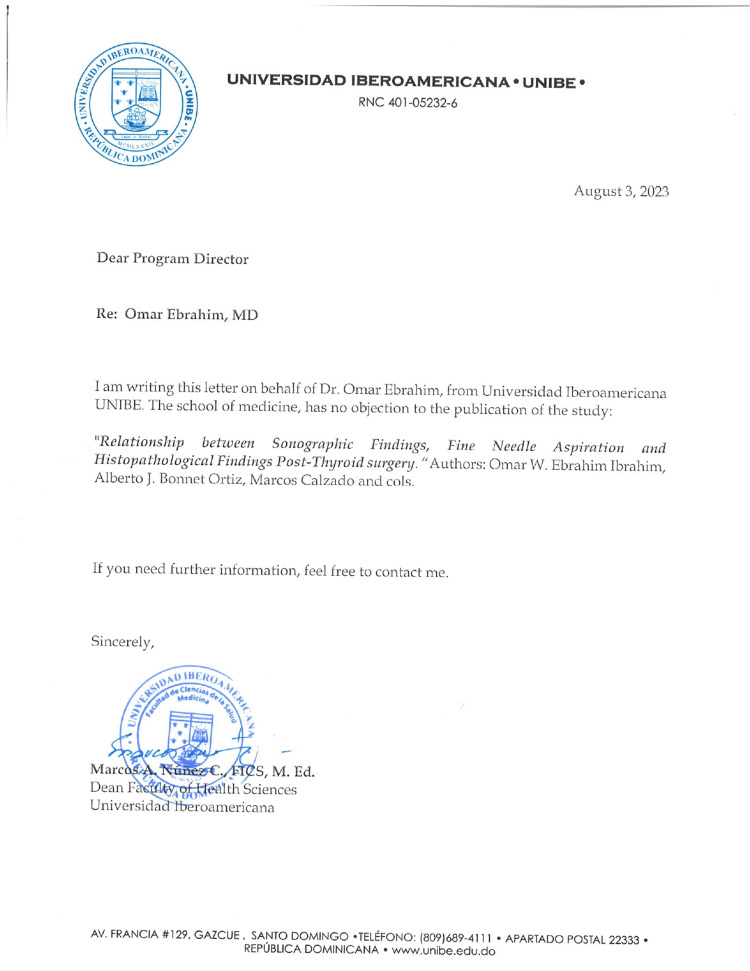
Authorization for publishing This document has been uploaded to confirm authorization as an original text adaptation for publishing
